# Socioeconomic Status Is Associated With Antibody Levels Against Vaccine Preventable Diseases in the Netherlands

**DOI:** 10.3389/fpubh.2018.00209

**Published:** 2018-07-27

**Authors:** Joske Hoes, Anna G. C. Boef, Mirjam J. Knol, Hester E. de Melker, Liesbeth Mollema, Fiona R. M. van der Klis, Nynke Y. Rots, Debbie van Baarle

**Affiliations:** ^1^Centre for Epidemiology and Surveillance of Infectious Diseases, Centre for Infectious Disease Control, National Institute for Public Health and the Environment, Bilthoven, Netherlands; ^2^Centre for Immunology of Infectious Diseases and Vaccines, Centre for Infectious Disease Control, National Institute for Public Health and the Environment, Bilthoven, Netherlands; ^3^Laboratory for Translational Immunology, Department Immunology, University Medical Centre Utrecht, Utrecht, Netherlands

**Keywords:** vaccine preventable diseases, immunoglobulin G, socioeconomic status, antibody level, cytomegalovirus

## Abstract

**Background:** We investigated whether low socioeconomic status (SES), which is associated with reduced health and life expectancy, might play a role in increased risk for infectious diseases. Therefore, we explored the association between SES and immunoglobulin G (IgG) levels against various pathogens.

**Methods:** We analyzed the association between SES [educational level and net household income (NHI)] and serum IgG concentration against measles, mumps, rubella, varicella, Haemophilus influenzae type B (HiB), pneumococcus, meningococcus serogroup C (MenC), and cytomegalovirus (CMV) collected within a national cross-sectional serosurvey (2006/2007) using linear regression analyses among non-vaccinated individuals.

**Results:** Higher educational level was associated with higher IgG concentrations against measles (GMC ratio 1.34, 95% CI 1.18–1.53) and rubella (1.13, 1.02–1.25) compared to low education level. In contrast, higher education level was associated with lower IgG concentrations against pneumococcus (0.78, 0.70–0.88), MenC (0.54, 0.44–0.68), and CMV (0.23, 0.18–0.31) compared to low education level. This pattern was also evident when NHI was used as SES indicator.

**Conclusion:** Our study suggests that socioeconomic status is associated with antibody levels in a pathogen-dependent manner. The results suggest that differences in serological response upon infection or differences in exposure might be involved in the variation in IgG levels between SES groups.

## Introduction

Socioeconomic status (SES) is a well-known determinant for health status and lower life expectancy. SES is often determined by income or educational level and forms an important factor in health studies (1, 2). While a lot of research focused on the relation between SES and chronic diseases, only few studies have investigated the association of SES with infectious diseases. Several studies have shown that individuals with a lower SES have a higher risk of being cytomegalovirus (CMV) seropositive (3–5), a prevalent betaherpesvirus which has been suggested to be associated with enhanced aging of the immune system. This finding was also confirmed by a modeling study on SES in relation to infection control, which showed a decreasing CMV antibody response with increasing SES (6). A recent study by Aiello et al. indeed suggests enhanced aging of the immune system in individuals with low SES (7). Immune responses play an important role in the outcome of infections and differ between individuals. Diversity in immune responses between individuals is attributable to genetics, age, sex, and the environment (8). Perhaps SES is such an environmental influence affecting immunity, and thereby may influence exposure to and infection with pathogens. In addition, exposure to environmental risk factors such as air pollution and crowding is thought to be enhanced in individuals with low SES compared to high SES (9). Whether the characteristics of low SES individuals as described above contribute to altered immunity to encountered pathogens as measured by immunoglobulin G (IgG), and whether this may reflect their vulnerable health status, is unknown.

Since little is known about the association between SES and naturally derived antibody levels against pathogens, this study aimed to explore the association between SES and IgG levels against multiple (vaccine preventable) infectious disease causing agents. Presence of antibodies can be considered indicative for exposure to or infection with the pathogen. To provide an overview and to examine differences between pathogens, IgG levels against measles, mumps, rubella, varicella zoster virus (VZV), *Haemophilus influenzae* type B (HiB), *Streptococcus pneumoniae* (pneumococcus), *Neisseria meningitidis* (meningococcus) serogroup C (MenC), and CMV were studied using data from a national serosurvey conducted in the Netherlands in 2006-2007 (Pienter2 study). Vaccinations against all these respiratory transmitted pathogens were included in the National Immunization Program (NIP) of the Netherlands during Pienter2, except for varicella and CMV (10). As we wanted to study merely the effect of SES on natural infection, we only included participants aged 40–79 years during the serosurvey, as they were not yet immunized through the NIP and the studied IgG levels are thus reflecting immunity acquired through natural exposure to the pathogen.

## Materials and methods

### Study design

The Pienter2 study (P2) is a Dutch population-wide cross-sectional serosurvey conducted between February 2006 and June 2007, aiming to collect data at a national level on antibody levels against pathogens that are targeted in the NIP. An extensive study description can be found elsewhere (11, 12). In short, the Netherlands were divided in five regions from which 40 municipalities (eight from each region) were randomly chosen, taken into account the number of inhabitants, to participate in the study. During the study, 19781 participants 0–79 years of age were invited to fill in a questionnaire and to donate a blood sample. This resulted in 6,348 (32%) completed questionnaires with a supplementary blood sample in the national sample including an oversampling of non-Western migrants. The P2 project was approved by the medical ethics testing committee of the foundation of therapeutic evaluation of medicines (METC-STEG) in Almere (clinical trial number; ISRCTN 20164309). Every participant signed an informed consent prior to study participation. All participants from the national sample (including oversampled migrants) who were 40–79 years of age were included in the current analyses (*n* = 2,743).

### Laboratory methods

Serological testing for antibodies against the different pathogens was performed with a fluorescent bead-based multiplex immunoassay (MMRV-MIA) using Luminex technology for simultaneous detection of measles, mumps, rubella, and varicella antibodies (13). For HiB, pneumococcus, and MenC antibody concentrations were determined likewise in separate assays (14, 15). Thirteen pneumococcal serotypes were analyzed in this study (i.e., serotype 1, 3, 4, 5, 6A, 6B, 7F, 9V, 14, 18C, 19A, 19F, and 23F). For CMV an ELISA kit, ETI-CYTOK-G PLUS (DiaSorin, Saluggia, Italy), was used with an upper detection limit of 10 IU/mL (3). Measurements above this threshold were set at 10 IU/mL (*n* = 149). IgG concentrations against all pathogens were determined and calibrated against internationally generally accepted standards, such as the World Health Organization (WHO) cut-offs.

### Variables

For the measurement of socioeconomic status, the highest completed level of education and the net household income per month (NHI) were used as SES indicators. Educational level was divided in the categories low (i.e., no or primary education), intermediate (i.e., junior technical school, lower general or intermediate vocational secondary education), and high (i.e., higher vocational or higher general secondary education, pre-university or university education) (12). For NHI, low SES was defined as <€1,150, intermediate SES as €1,151–€3,050, and high SES as >€3,050 per month. As educational level and NHI are related but are not considered to be interchangeable, we decided to use both in separate analyses and in a combined analysis (16). Other characteristics that might be related to SES include age, sex, and ethnicity (16, 17). Therefore, the analyses were conducted with all ages combined and stratified by age group (i.e., 40–49, 50–59, 60–69, and 70–79 years). We also performed unadjusted and adjusted analyses, taking into account age category, sex, and ethnicity (Dutch vs. non-Dutch).

### Data analyses

We only included participants for whom all determinants and all antibody response data were available, and who were most likely not vaccinated against the selected pathogens (based on available vaccination data). Geometric mean concentrations (GMC) with 95% confidence intervals (CI) were calculated per subgroup based on the categorization of educational level or NHI, and age. The survey design with five strata (regions) and 40 clusters (municipalities) was taken into account in all analyses, which corrected the standard error of the estimates.

Linear regression analyses were performed to study the association between SES and the logarithmically transformed antibody concentration against the different pathogens. Separate linear regression analyses were conducted between NHI and educational level and pathogen-specific antibody level, for all ages combined and stratified by age groups. In the crude model, only the SES variable was included, while the adjusted model included age category, sex, and ethnicity. The interaction with age category was tested as well. We also performed a similar analysis of educational level and NHI combined, in which we only compared participants who were in the high category for both educational level and NHI to participants who were in the low category for educational level and NHI. The same was done for people who were in the intermediate category for educational level and NHI (vs. low category). In all analyses, we present the results as GMC ratios comparing high and intermediate SES to low SES.

In addition to the separate pneumococcal serotype specific regression models, a generalized estimation equation model (GEE) was applied to obtain an overall estimate of the GMC ratio across all pneumococcal serotypes with corresponding 95% CI, as the strength of the association with SES did not differ greatly between serotypes. The model included the SES determinant, and serotype for the crude estimates, combined with age category, sex, and ethnicity for the adjusted estimates. An exchangeable working correlation structure was used. *P* < 0.05 were considered significant. All analyses were conducted in SAS 9.4.

### Validation of study results

To validate our results, we performed the same analyses using data from the Pienter1 study (P1), conducted between October 1995 and December 1996, which has a study design similar to P2 [details are described elsewhere (18)]. 15,189 inhabitants were invited to participate, of whom 8,539 (56%) completed the questionnaire and donated a blood sample. For the validation analyses in this project, 3,609 participants aged 40–79 years with questionnaires and blood samples available were included in the analyses, without oversampling of Non-Western migrants. In P1, only educational level as SES indicator was available. Antibody concentrations against HiB, MenC, and pneumococcus were determined in a similar manner in P1 and P2 (15, 19). For measles, mumps, rubella, and varicella antibodies were determined with ELISAs instead of MIAs (20–23), which showed good correlation between the assays (13). IgG levels against CMV were not determined. IgG levels were not available for all participants, leaving 629 (varicella) to 3,575 samples (measles, mumps, rubella) available for analysis (number of samples analyzed per pathogen are listed in **Figure 4**).

## Results

General characteristics of participants from P2 who had blood and questionnaires available are listed in Table 1 (*n* = 2,743). The majority of the study population was Dutch (>80%). Seventy-one participants were excluded because they received (part of a) MMR vaccination, 510 participants were excluded as no information on NHI was available, and 41 participants were excluded, as educational level was unknown. This left us with 2,172 participants for the NHI analyses, and 2,627 participants for the educational level analyses.

**Table 1:** Descriptive characteristics of participants from the Pienter2 national sample 40–79 years, with blood sample and questionnaire available (*N* = 2,743).

**Characteristic**	***N* (%)**
**SEX**	
Male	1,248 (45.5)
Female	1,495 (54.5)
**AGE (YEARS)**	
40–49	641 (23.4)
50–59	713 (26.0)
60–69	797 (29.0)
70–79	592 (21.6)
**EDUCATIONAL LEVEL^a^**	
Low	373 (13.6)
Intermediate	1,442 (52.6)
High	887 (32.3)
Missing	41 (1.5)
**NET HOUSEHOLD INCOME**	
Low (<€1,150,-)	423 (15.4)
Intermediate (€1,151,- to €3,050,-)	1,368 (49.9)
High (>€3,050,-)	442 (16.1)
Missing	510 (18.6)
**ETHNICITY**	
Dutch	2,228 (81.2)
Non-Dutch	515 (18.8)
**URBANIZATION RATE**	
Very high (>2,500 addresses per km^2^)	565 (20.6)
High (1,500–2,500 addresses per km^2^)	1,238 (45.1)
Moderately high (1,000–1,500 addresses per km^2^)	353 (12.9)
Low (500–1,000 addresses per km^2^)	272 (9.9)
Very low (<500 addresses per km^2^)	315 (11.5)
**NUMBER OF PEOPLE IN HOUSEHOLD**	
1–2 persons	1,840 (67.1)
≥3 persons	863 (31.5)
Missing	40 (1.4)
**CHILD ≤ 4 YEAR IN HOUSEHOLD**	
Yes	69 (2.5)
No	2,674 (97.5)
**PROFESSION WITH CHILDREN**	
Yes	381 (13.9)
No	2,361 (86.1)
**ANY MMR VACCINATION RECEIVED (WHOLE OR PART)**	
Yes	71 (2.6)
No	2,672 (97.4)

a *Low educational level was defined as no education or primary education, intermediate educational level was defined as junior technical school, lower general or intermediate vocational secondary education, and high educational level was defined as higher vocational or higher general secondary education, pre-university or university education (according to CBS)*.

**Figure 1:**
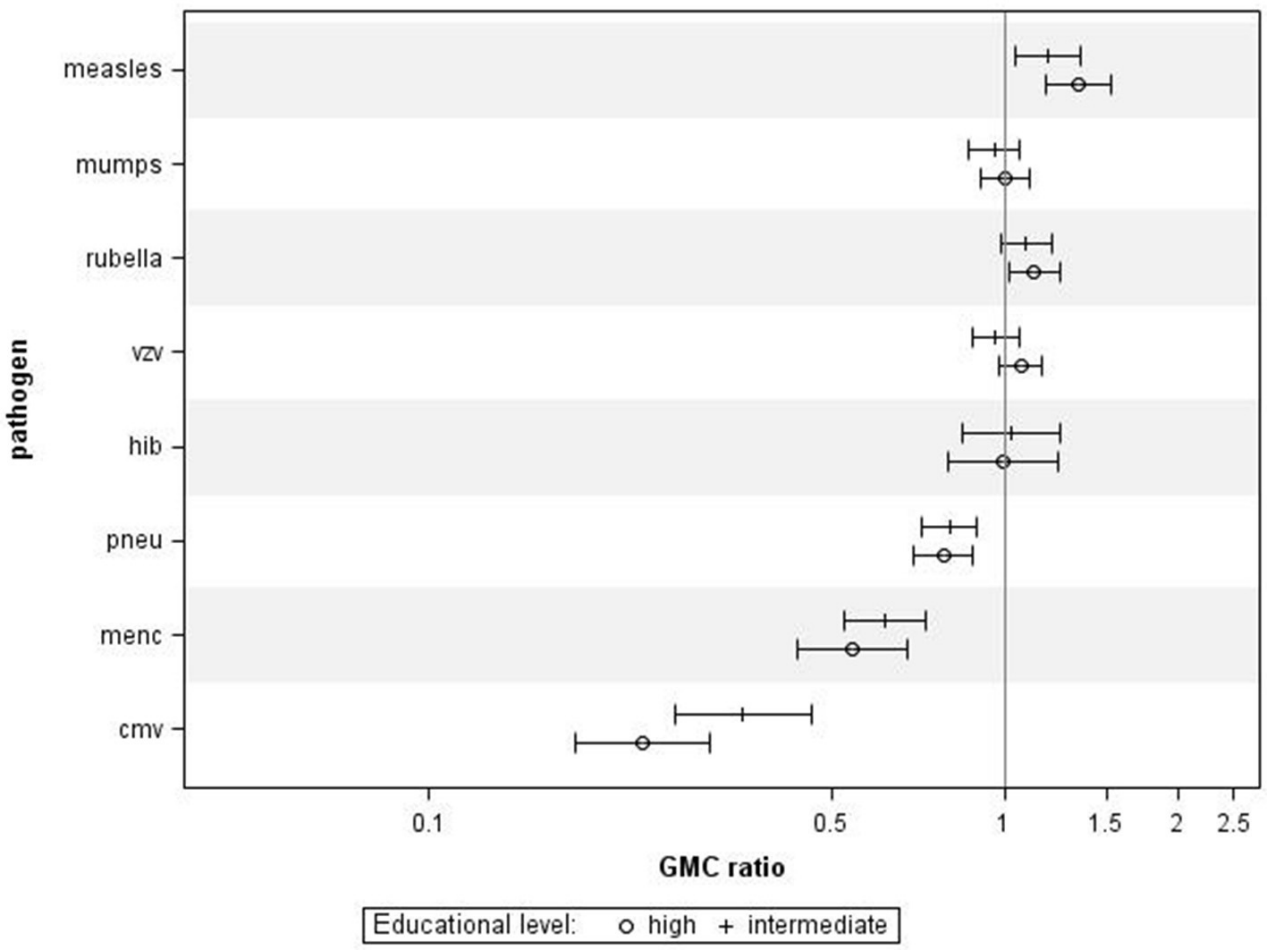
Crude geometric mean concentration ratios per pathogen of high and intermediate educational level vs. low educational level with corresponding 95% C.I. *N* = 2,627.

When combining all age groups, higher educational level was significantly associated with higher IgG against measles (GMC ratio 1.34, 95% CI 1.18–1.53, *p* < 0.0001) and rubella (GMC ratio 1.13, 95% CI 1.02–1.25, *p* = 0.02) compared to lower educational level, (Figure 1). High educational level was not significantly associated with IgG levels against mumps, varicella, and HiB. In contrast, high educational level was significantly associated with lower IgG levels against MenC (GMC ratio 0.54, 95% CI 0.44–0.68, *p* < 0.0001), and pneumococcus (GMC ratio 0.78, 95% CI 0.70–0.88, *p* < 0.0001), compared to lower educational level. Serotype-specific pneumococcal GMC ratios for high educational level vs. low educational level for all ages combined ranged between 0.65 (95% CI 0.53–0.79, *p* < 0.0001) for serotype 6A and 1.02 (95% CI 0.86–1.20, *p* = 0.841) for serotype 3. Also for CMV higher educational level was significantly associated with lower IgG levels compared to lower educational level (GMC ratio 0.23, 95% CI 0.18–0.31, *p* < 0.0001). GMC IgGs against all pathogens were calculated per age group and educational level, and are included in Supplementary Figure 1. All associations were fairly similar for the different age groups, although for measles, pneumococcus, and MenC the associations were not significant in all age groups (Supplementary Figure 3). Interaction with age categories showed significant results for measles (*p* = 0.045), rubella (*p* = 0.007), and HiB (*p* = 0.006), indicating different strengths of the association across the age groups for these pathogens.

**Figure 2:**
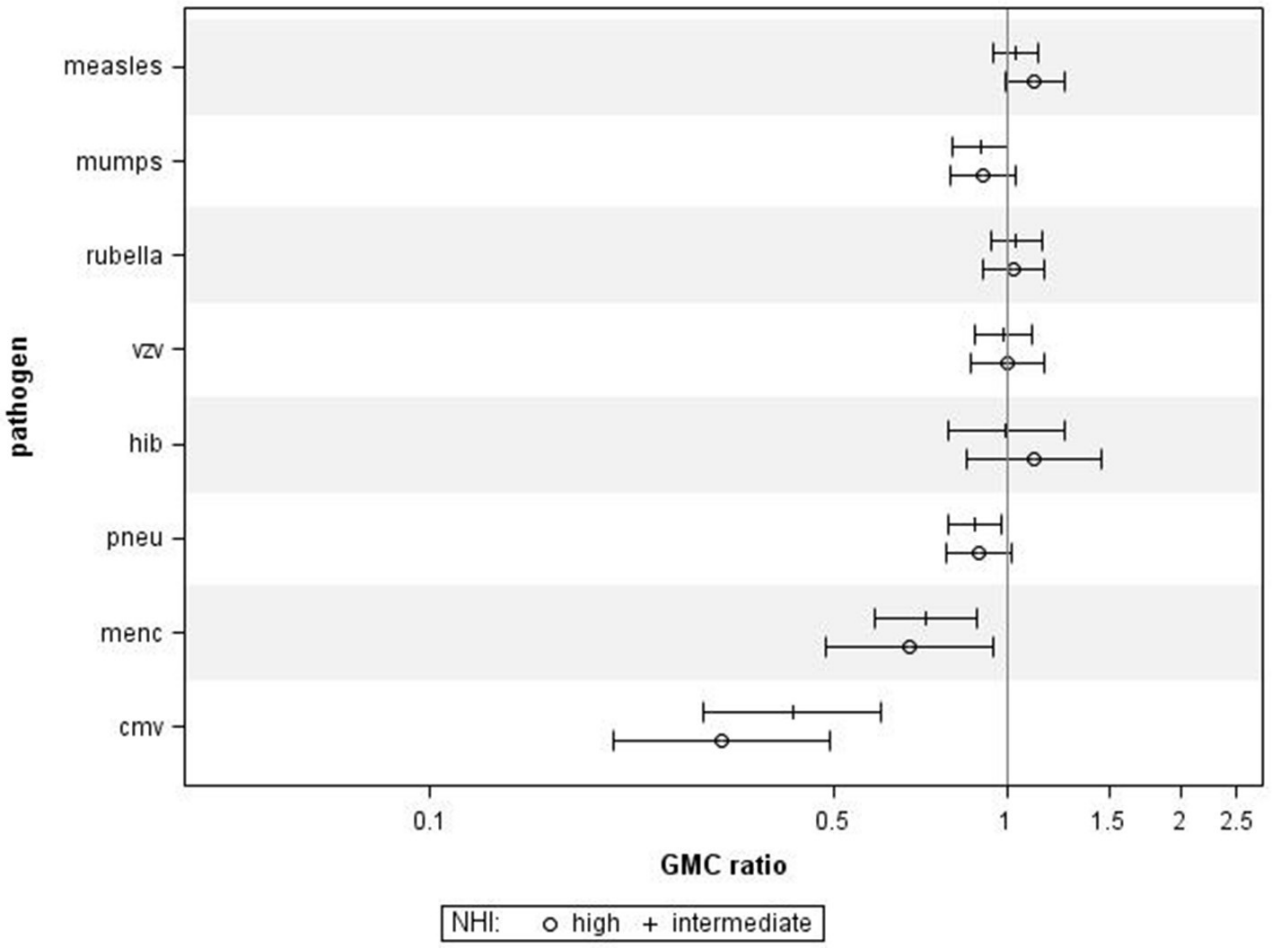
Crude geometric mean concentration ratios per pathogen of high and intermediate net household income (NHI) level vs. low NHI with corresponding 95% C.I. *N* = 2,172.

Using NHI as SES indicator for all ages combined revealed that higher NHI was borderline significantly associated with higher IgG against measles (GMC ratio 1.12, 95% CI 0.99–1.26, *p* = 0.071) (Figure 2). High NHI was not significantly associated with IgG levels against mumps, rubella, varicella, HiB, and pneumococcus. High NHI was significantly associated with lower IgG levels against MenC (GMC ratio 0.68, 95% CI 0.49–0.94, *p* = 0.023), and CMV (GMC ratio 0.32, 95% CI 0.21–0.49, *p* < 0.0001) compared to low NHI. Serotype-specific pneumococcal GMC ratios for high NHI compared to low NHI for all ages ranged between 0.73 (95% CI 0.59–0.90, *p* = 0.004) for serotype 9V and 1.11 (95% CI 0.92–1.35, *p* = 0.269) for serotype 3. GMC IgGs against all pathogens were calculated per age group and NHI category, and are included in Supplementary Figure 2. The observed associations were fairly similar for the different age groups (Supplementary Figure 4). Interactions with age category showed significant results for rubella (*p* = 0.009), and HiB (*p* = 0.008). Moreover, both in the educational level and NHI analysis a dose response relationship was observed for almost all pathogens, with high SES differing more from low SES in IgG GMC compared to the intermediate SES (*p*-values for trends are included in Table 2).

**Table 2:** *P*-values for trend across the socioeconomic status categories, education and net household income are analyzed separately.

	**Education**	**NHI**
Measles	<0.0001	0.1061
Mumps	0.6785	0.1768
Rubella	0.0929	0.7071
Varicella	0.0645	0.9963
HiB	0.8728	0.4721
MenC	0.0005	0.0477
Pneu	0.0005	0.1000
CMV	<0.0001	<0.0001

**Figure 3:**
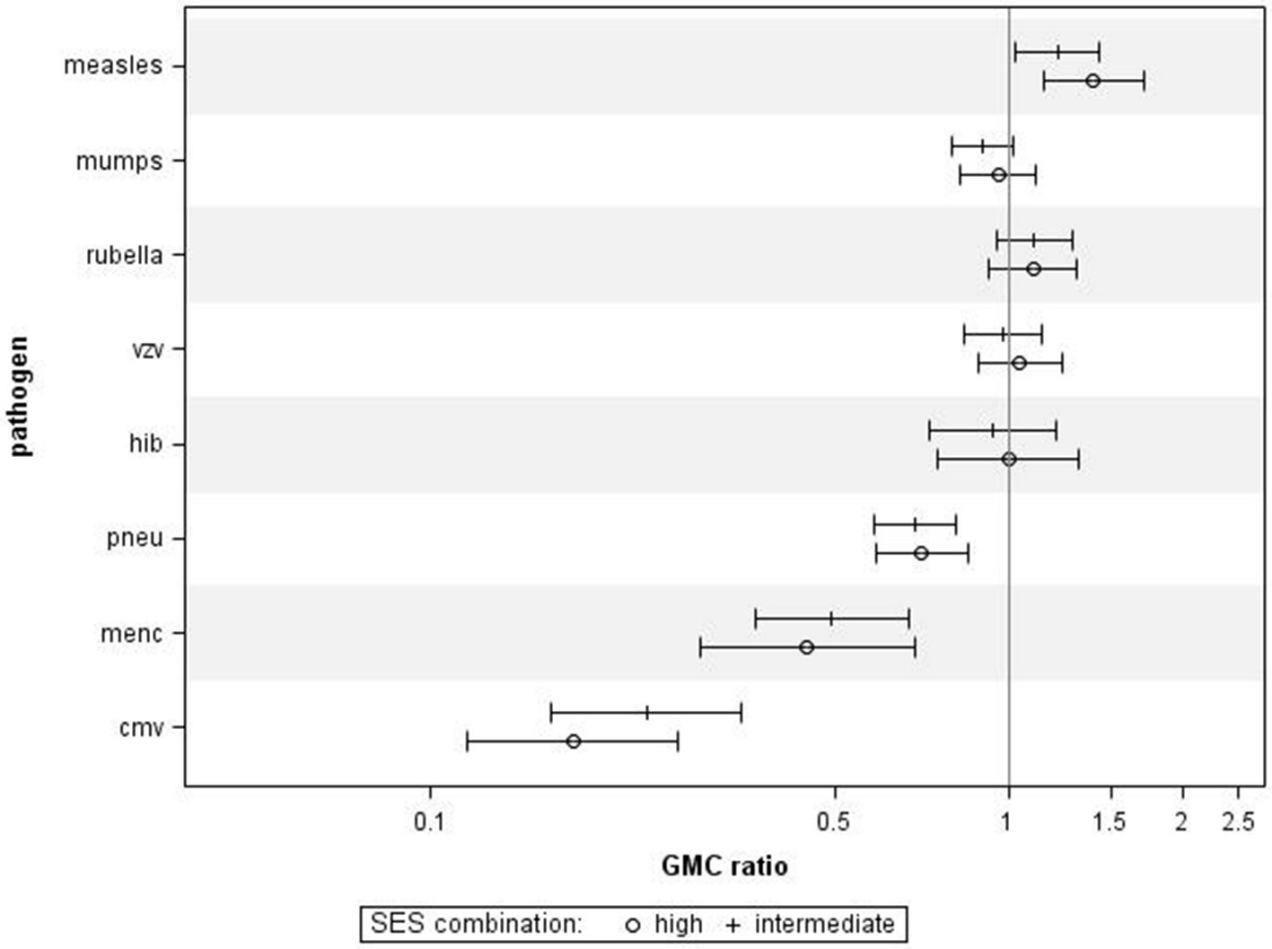
Crude geometric mean concentration ratios per pathogen of socioeconomic status (SES) combined. High and intermediate SES compared to low SES with corresponding 95% C.I. *N* = 1,256.

When performing a combined analysis of educational level and NHI, in which we compared participants who were in the low category for both educational level and NHI to participants who were in the high category for educational level and NHI, we observed similar associations (Figure 3). Adjusting for age category, sex, and ethnicity did not change the general pattern in all analyses (data not shown).

**Figure 4:**
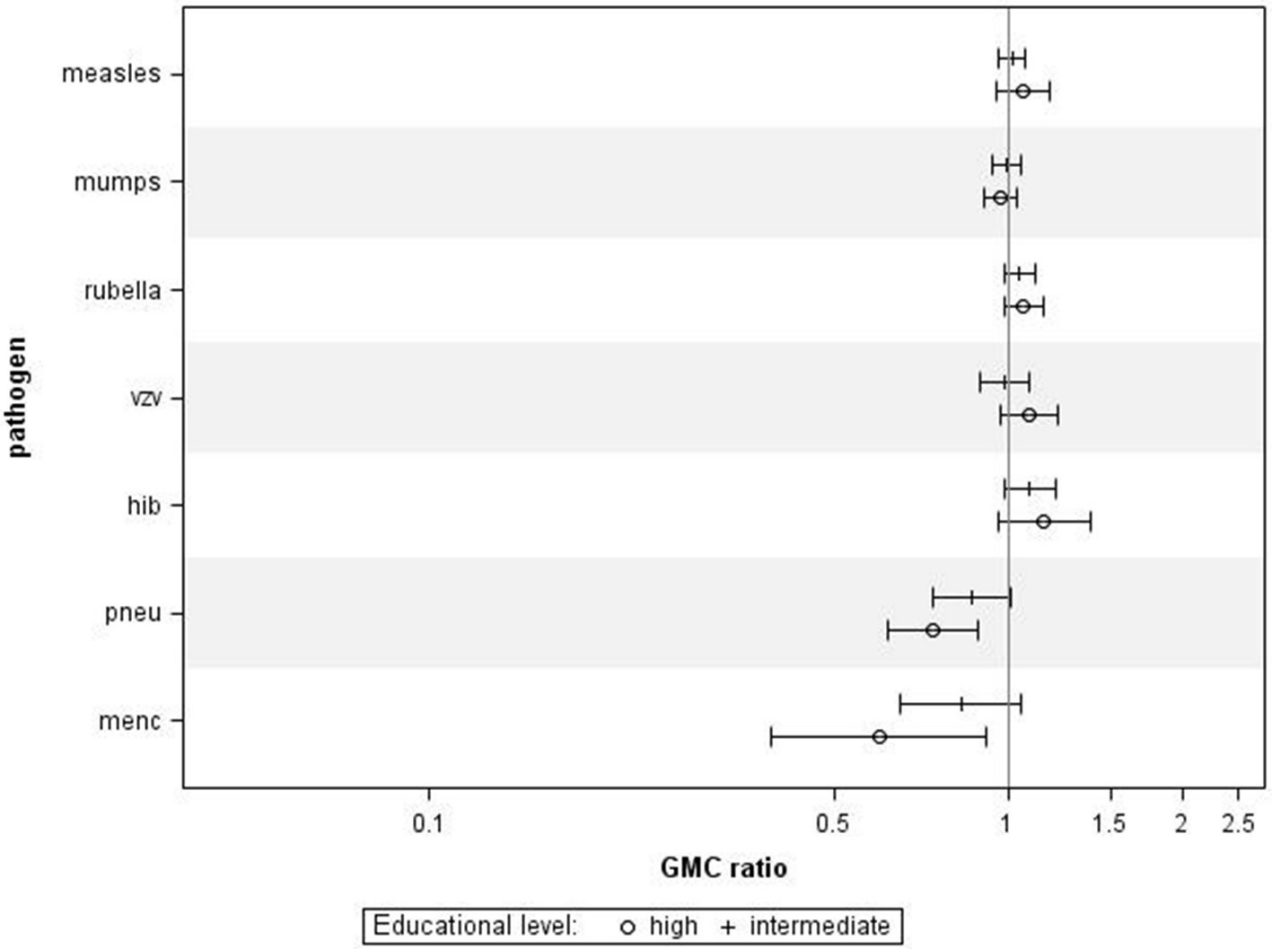
Validation in Pienter 1. Crude geometric mean concentration ratios per pathogen of high and intermediate educational level vs. low educational level with corresponding 95% C.I. *N* (measles, mumps, rubella) = 3,575, *N* (varicella) = 629, *N* (HiB) = 3,571, *N* (pneumo) = 723, *N* (MenC) = 749.

Finally, we also investigated the associations between educational level and IgG levels against the antigens tested in the P1 study, performed 10 years prior to the P2 study. For all ages combined, we observed similar associations between educational levels and IgG concentrations against most pathogens (Figure 4), although, educational level was not significantly associated with IgG levels against measles, mumps, rubella, varicella, and HiB. Higher educational level was significantly associated with lower IgG levels against MenC (GMC ratio 0.60, 95% CI 0.39–0.92, *p* = 0.024) and pneumococcus (GMC ratio 0.74, 95% CI 0.62–0.89, *p* = 0.001) compared to lower educational level.

## Discussion

In this study, the association between socioeconomic status and naturally derived IgG antibody concentrations as a measure for infectious disease induced immune response across different pathogens was explored. We investigated both educational level and income as separate SES indicators, and found for both criteria that, in general, people with lower SES have higher antibody levels against pneumococcus, MenC, and CMV, and lower IgG levels against measles. No association was found between SES and IgG level against mumps, rubella, varicella, and HiB. After adjusting for age category, sex, and ethnicity, this pattern remained evident.

The directions of the association observed in our results are, mostly, supported by earlier research; The Third National Health and Nutrition Examination Survey (NHANES III, 1988–1994) in the United States showed that less years of education were associated with higher antibody levels against CMV in all age groups, as was a lower family income (24). Data from another NHANES study (1999–2004), also indicated that individuals with “less than or equal to high school” educational level, had lower odds on being measles seropositive (25). However, such associations were not found for rubella (26). There are studies indicating that the incidence of HiB disease is higher among socially disadvantaged populations (27, 28). However, we were not able to confirm this in our study through different IgG levels between SES groups. Furthermore, community acquired pneumonia (CAP), which can be caused by pneumococcus, is thought to be associated with lower SES (29), and also the risk of invasive meningococcal disease (IMD) in children younger than 15 years of age is inversely related to SES factors such as parental educational level and crowding (30). Also Cleary and colleagues found an increased risk of meningococcal carriage with decreasing SES (31). This suggests that lower antibody levels against these bacteria may indicate less carriage of these bacteria and thereby less risk of developing CAP or IMD.

Interestingly, the association between SES and antibody levels was clearly different for the various pathogens. Differences in the nature of the pathogen or differences in carriage and exposure might be involved in the different outcomes. Pneumococci, meningococci, and HiB are characterized by (widespread) carriage among children and adolescents, while CMV is increasingly present with increasing age (4, 32). Measles, mumps, rubella and varicella are viruses inducing typical children's diseases after which the pathogen is cleared, except for varicella zoster virus, which remains latently present. In addition, there is less circulation of these pathogens among the adult population, as the antibodies provide protection for a long time (33–36).

Therefore, higher antibody responses to infections such as pneumococcus and MenC (32), might reflect higher exposure to these pathogens in low SES individuals. Moreover, pathogen carriage and exposure might be related to SES factors as well; for example, air pollution, crowding, or knowledge about (hand) hygiene might be involved in the frequency and intensity of carriage and exposure, which can in turn be reflected in IgG levels against such pathogens. The absence of an association for HiB might be explained by a lower carriage rate compared to pneumococcus and MenC, and less disease. This results in less circulation and fewer differences in exposure and IgG levels. On the other hand, IgG levels against typical children's diseases (i.e., measles, mumps, rubella, and varicella) are more likely to reflect the primary response evoked by exposure during childhood, as there is little exposure later in life. As there are no differences in seroprevalence between the SES groups for the viruses (all about 99%), primary response seems important here. On the other hand, for CMV we see a steep increase of seroprevalence with decreasing SES, which is indicative for more exposure to the pathogen, perhaps as a result of difference in hygienic measures or contact with children. However, more research is needed to study these hypotheses.

Several elements of this study support the robustness of our findings. First, the SES indicators that were used (i.e., educational level and NHI) showed a similar effect on the antibody levels, although the differences between SES categories were less outspoken when using NHI (which could have been caused by smaller numbers). Educational level might better reflect SES in early life and provides the foundation and knowledge for healthy behavior, while NHI is a better representative of recent socioeconomic status and the amount of money people can invest in their health through nutrition or housing later in life (37). Including both indicators in the study separately provided us with a more reliable result as the general pattern was quite consistent across the indicators. Importantly, a similar pattern was observed in the P1 study using only educational level as SES indicator, which validated our results in another study population. In addition, the results remained evident across age groups in both P1 and P2. Finally, a dose-response effect was observed for both the income and educational level, with (for most of the analyses) the high SES being more different from the low SES than the intermediate SES.

The methodological decision to include only participants with complete data in the analyses was based on the missing NHI data. As NHI is most likely to be missing not at random (MNAR), i.e., not depending on measured covariables but on the value of the variable itself, statistical solutions such as imputation are not reducing the bias (38). We decided to include only participants with all determinants available, realizing that we might be missing a specific set of people in the NHI analysis who did not want to provide the height of their income, which we assume is most likely to be rather low or very high. However, the fact that the same results were found when educational level or NHI was used as SES indicator is reassuring that the potential bias induced by the missing NHI data on the association is minimal.

Some studies use more SES categories or other SES variables such as poverty level or occupation (16). In the current study, we only included educational level and NHI as SES indicators, as no other SES information was available. The categories were based on the P2 studies (12), however, sometimes a fourth (education) category is included in health research. We decided not to do so, as the expected differences between categories were minor. Moreover, we analyzed the SES indicators separately and combined, as educational level and NHI are not interchangeable (16) and found similar results.

Our study included a large sample size from a national database, which enabled us to assess antibody levels in three SES categories. Limitations that should be considered include the cross-sectional design of the serosurvey, which restricted us in studying causal mechanisms. In addition, data on smoking and drinking habits were not available in P2, which can be considered a drawback. This information could have been relevant to consider, as these are specific lifestyle-related risk factors for invasive pneumococcal disease (IPD) and IMD (39, 40), and as it could perhaps have explained the effect of educational level we found. This also holds for other exposure related variables that might have explained the observed associations, which we did not have in the questionnaire or only for a limited number of people. Although we excluded individuals with documented or self-reported vaccination against measles and rubella, there might still be some individuals (in particular in the younger age classes) that were vaccinated, which could have affected the measured IgG levels. Finally, an important difficulty embedded in this study is what should and what should not be taken into account when studying SES. Socioeconomic status is an abstract phenomenon and educational level or income is only a proxy to study this construct (16). SES itself is probably not the causal factor, but other factors that are represented by or related to SES might cause differences in IgG responses after infection (37).

In conclusion, we showed that socioeconomic status is associated with naturally derived antibody levels and that this association is pathogen-dependent. The results suggest that differences in serological response upon infection or differences in exposure might be involved in the variation in IgG levels between SES groups. More research is needed to thoroughly establish this association and the mechanisms through which differences in antibody levels are induced, to investigate this association in vaccinated individuals, and to assess whether an association can also be established between SES and reported illness.

## Author contributions

DvB, NR, and MK designed the study. JH, AB, and MK analyzed the data. All authors interpreted the data. JH wrote the first draft of the manuscript. All authors critically reviewed and commented on the manuscript before agreeing with the final version.

### Conflict of interest statement

The authors declare that the research was conducted in the absence of any commercial or financial relationships that could be construed as a potential conflict of interest.
