# Supplementary material for: Socioeconomic Status Is Associated With Antibody Levels Against Vaccine Preventable Diseases in the Netherlands
Source: Front Public Health. 2018 Jul 27;6:209. doi: 10.3389/fpubh.2018.00209 (PMC6094970; doi:10.3389/fpubh.2018.00209)
Supplement: Supplementary file 1 [file Image_1.pdf]

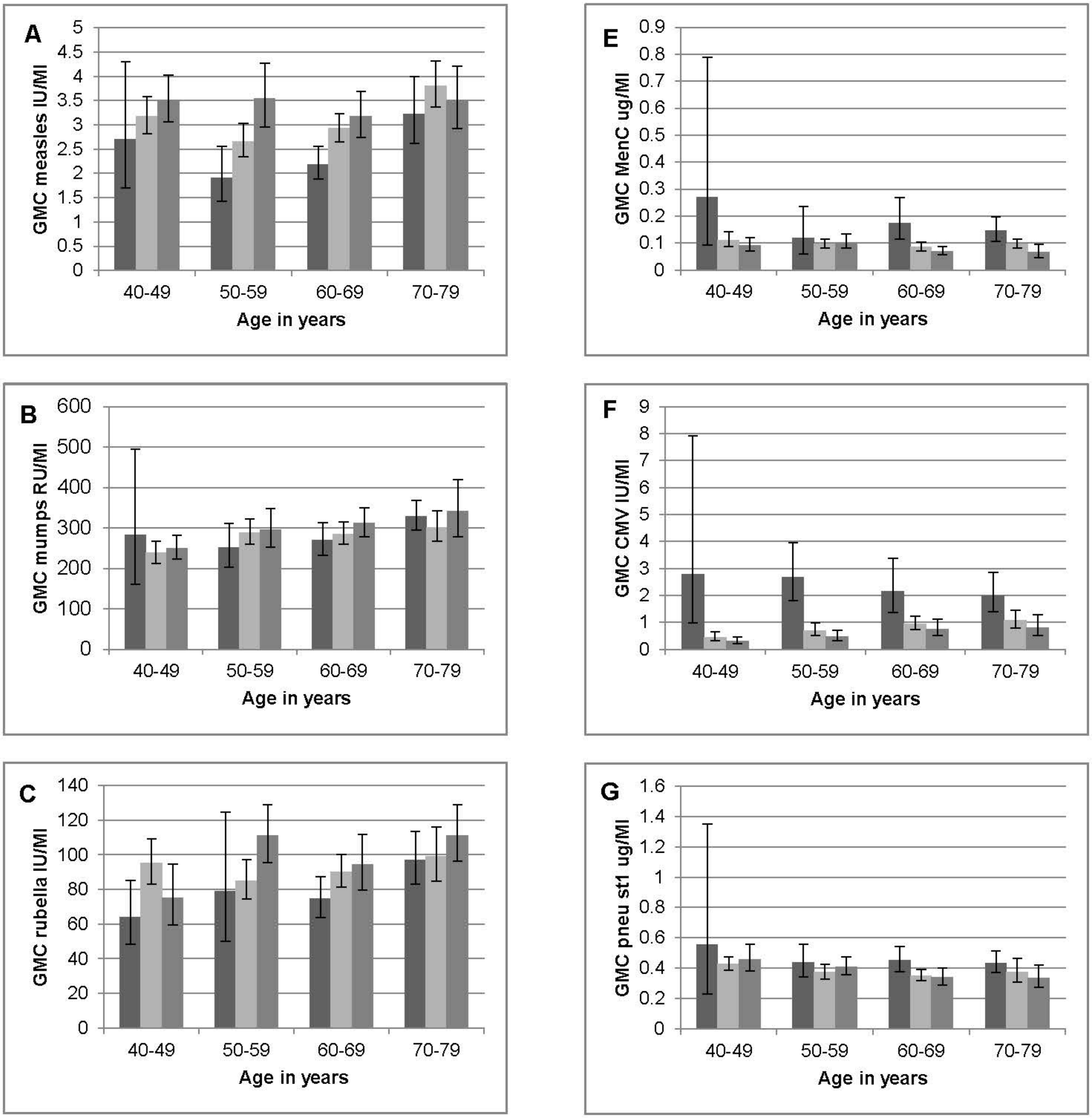

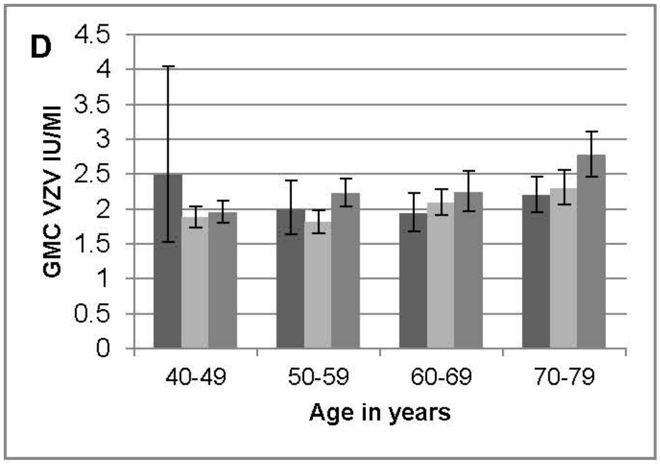

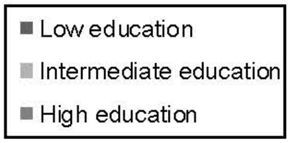

Supplementary Figure 1 - part 1. Age specific geometric mean concentrations with 95% CI by different levels of education. Note: different scales were used on the y-axis.

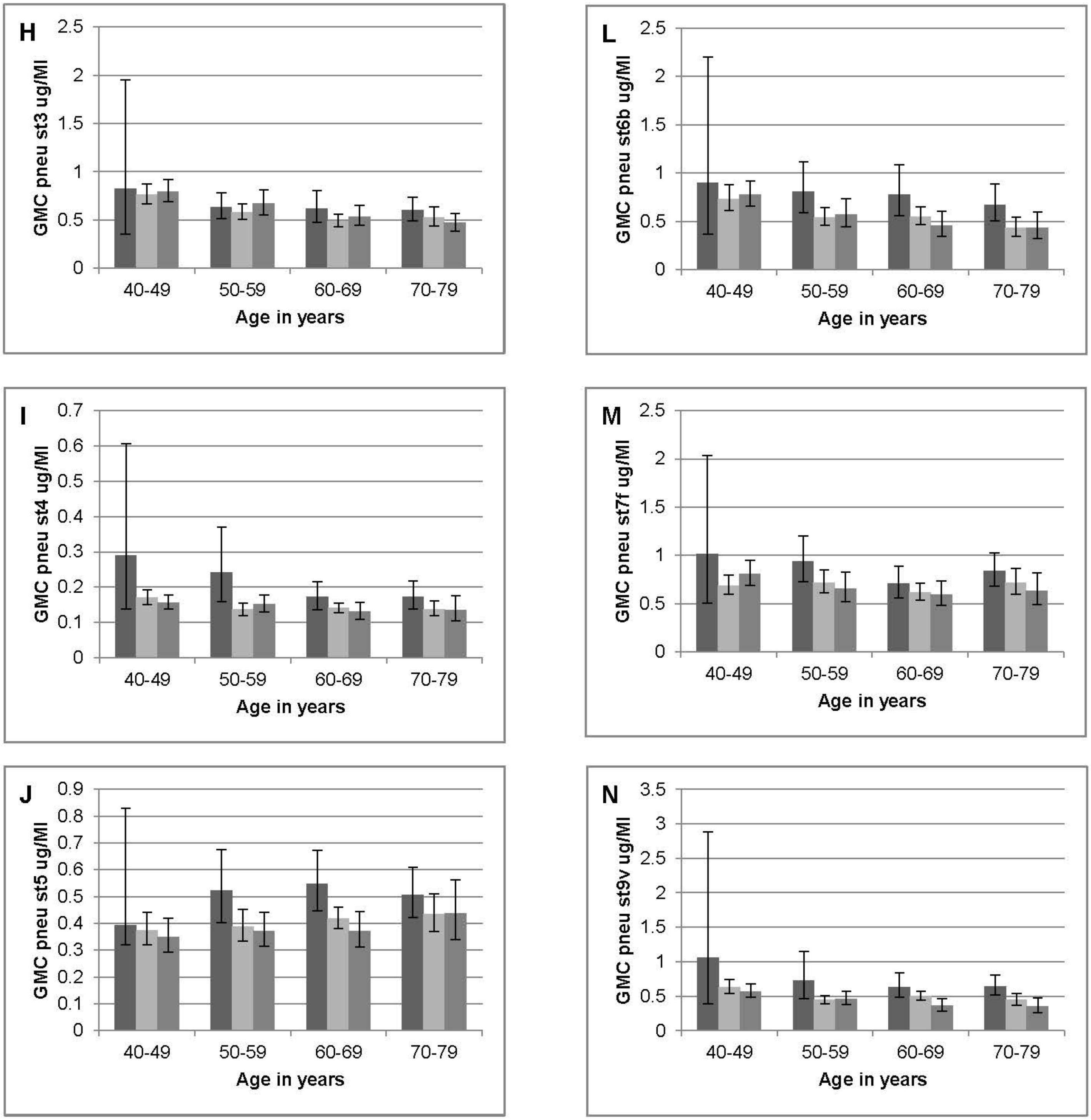

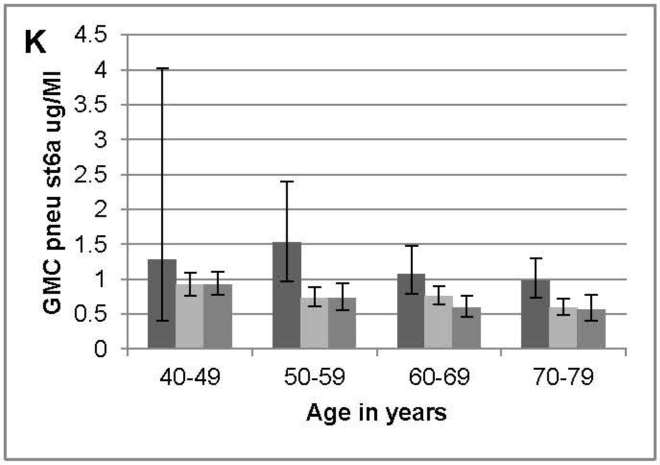

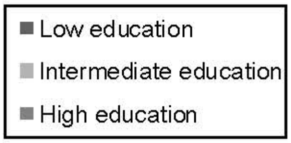

Supplementary Figure 1 - part 2. Age specific geometric mean concentrations with 95% CI by different levels of education. Note: different scales were used on the y-axis.

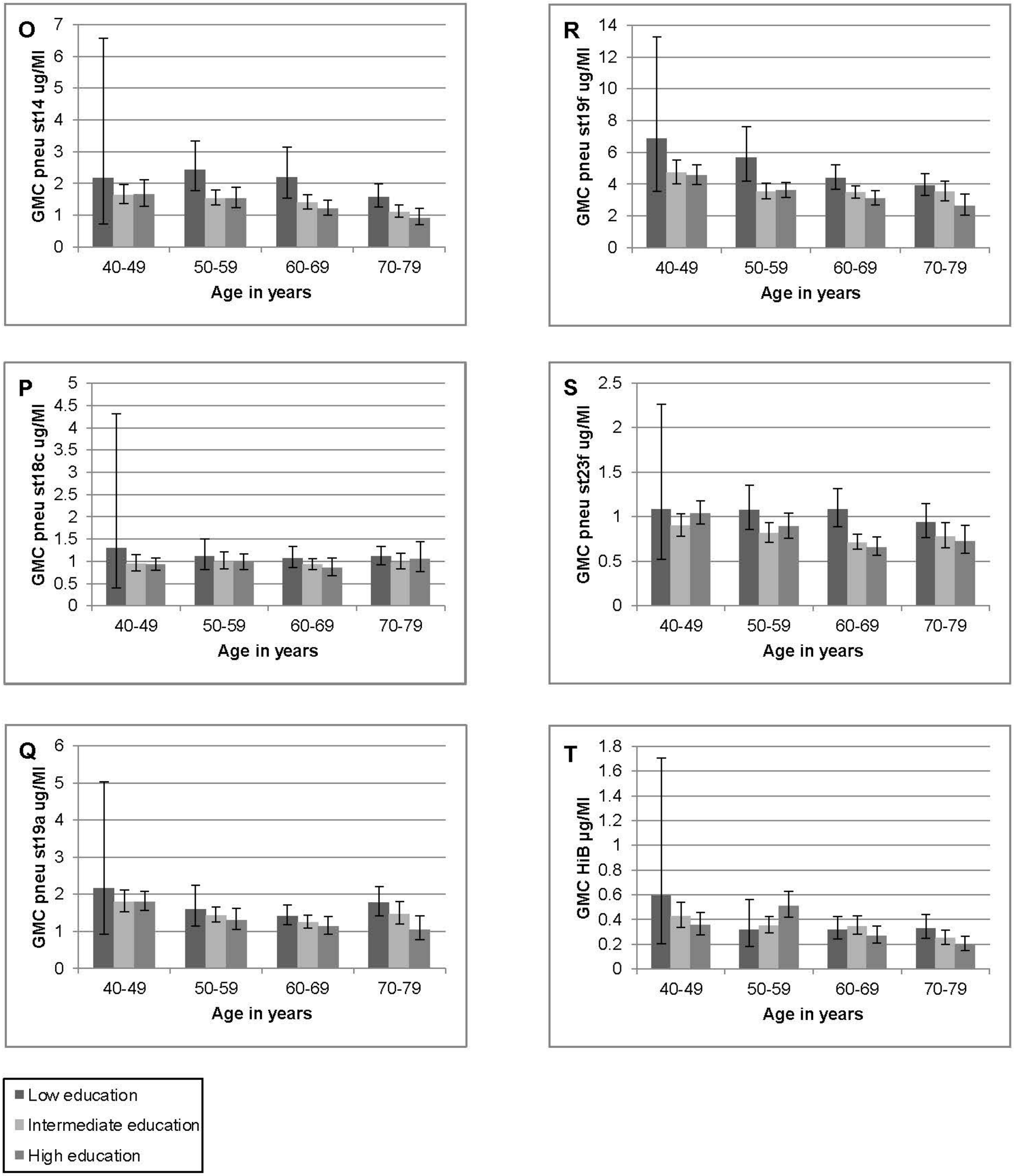

Supplementary Figure 1 - part 3. Age specific geometric mean concentrations with 95% CI by different levels of education. Note: different scales were used on the y-axis.
