# Supplementary figures and images for: Socioeconomic Status Is Associated With Antibody Levels Against Vaccine Preventable Diseases in the Netherlands
Source: Front Public Health. 2018 Jul 27;6:209. doi: 10.3389/fpubh.2018.00209 (PMC6094970; doi:10.3389/fpubh.2018.00209)

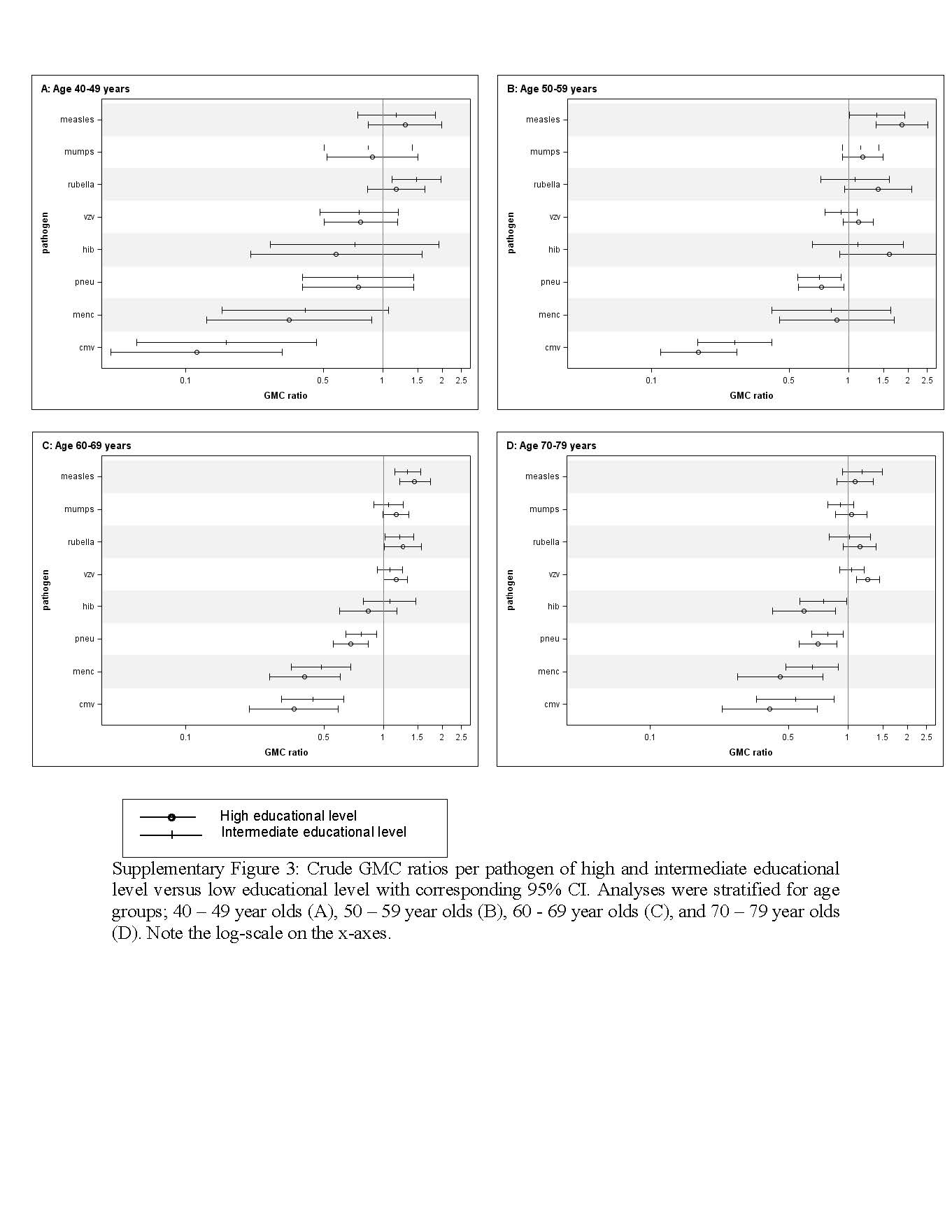

Supplement: Supplementary file 3 [file Image_3.jpeg]

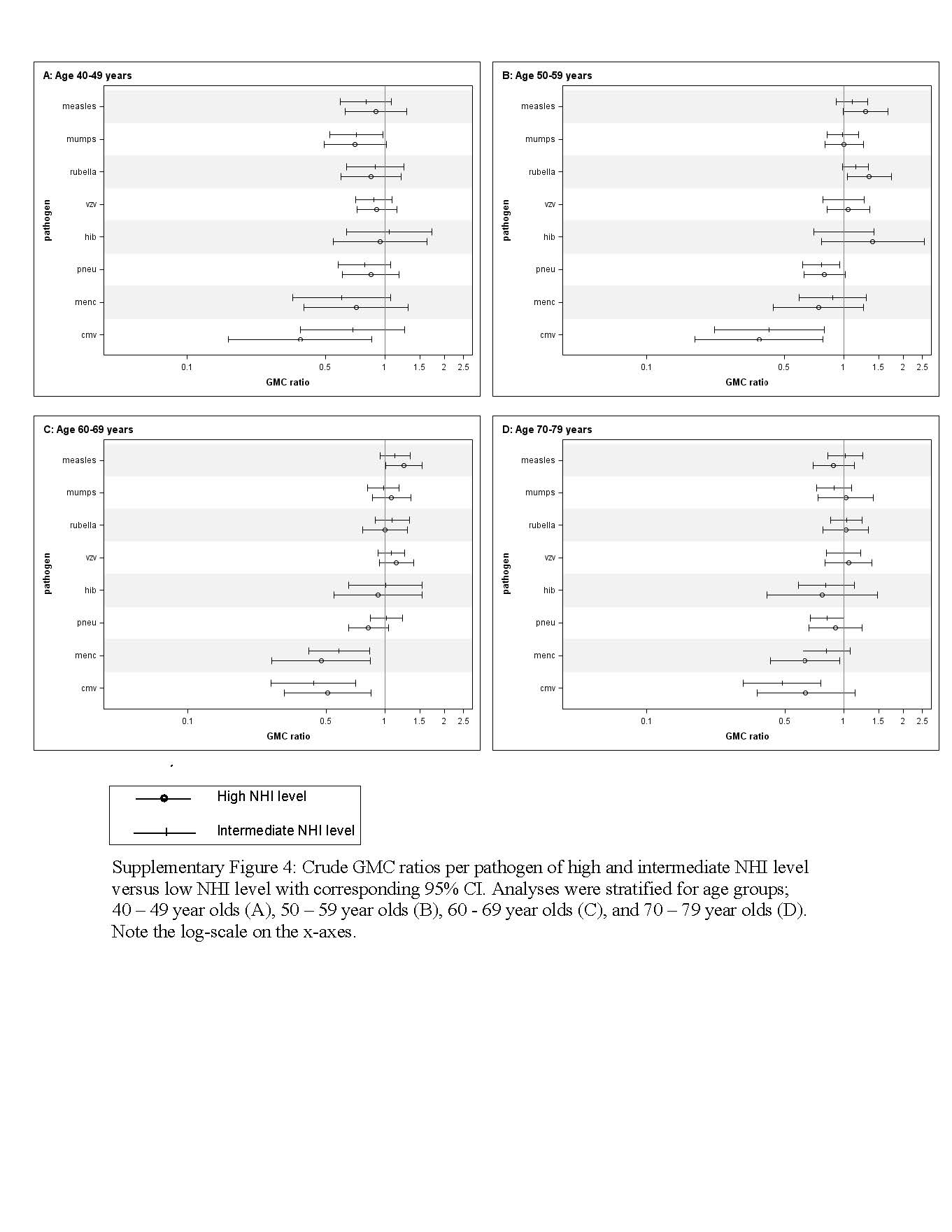

Supplement: Supplementary file 4 [file Image_4.jpeg]
